# International Consortium for Health Outcome Measurement Set of Outcomes That Matter to People Living With Inflammatory Arthritis: Consensus From an International Working Group

**DOI:** 10.1002/acr.23799

**Published:** 2019-11-29

**Authors:** Martijn A. H. Oude Voshaar, Zofia Das Gupta, Johannes W. J. Bijlsma, Annelies Boonen, Jeffrey Chau, Delphine S. Courvoisier, Jeffrey R. Curtis, Benjamin Ellis, Sofia Ernestam, Laure Gossec, Christine Hale, Jennifer Hornjeff, Katy Y. Y. Leung, Merav Lidar, Phillip Mease, Kaleb Michaud, Girish M. Mody, Mwidimi Ndosi, Christina H. Opava, Geraldo R. C. Pinheiro, Matthew Salt, Enrique R. Soriano, William J. Taylor, Maria J. H. Voshaar, Angelique E. A. M. Weel, Maarten de Wit, Nico Wulffraat, Mart A. F. J. van de Laar, Harald E. Vonkeman

**Affiliations:** ^1^ University of Twente Enschede The Netherlands; ^2^ International Consortium for Health Outcomes Measurement London UK; ^3^ University Medical Center Utrecht Utrecht The Netherlands; ^4^ Maastricht University Medical Centre Maastricht The Netherlands; ^5^ Hong Kong Psoriatic Arthritis Association Hong Kong China; ^6^ University Hospitals of Geneva and University of Geneva Geneva Switzerland; ^7^ University of Alabama at Birmingham; ^8^ Imperial College Healthcare NHS Trust London UK; ^9^ Karolinska Institutet Stockholm Sweden; ^10^ Sorbonne Université and Pitié Salpêtrière Hospital AP‐HP Paris France; ^11^ Lockton Dunning Benefits Dallas Texas; ^12^ Columbia University Medical Centre New York New York; ^13^ Singapore General Hospital, Duke‐NUS Medical School Singapore; ^14^ Sheba Medical Centre Tel‐Hashomer Israel; ^15^ Providence St. Joseph Health System University of Washington Seattle; ^16^ University of Nebraska Medical Center Omaha, and the National Databank for Rheumatic Diseases Wichita Kansas; ^17^ University of KwaZulu‐Natal Durban South Africa; ^18^ University of the West of England Bristol UK; ^19^ Universidade do Estado do Rio de Janeiro Rio de Janeiro Brazil; ^20^ Instituto Universitario Hospital Italiano de Buenos Aires Buenos Aires Argentina; ^21^ University of Otago Wellington New Zealand; ^22^ Maasstad Hospital Rotterdam The Netherlands; ^23^ VU University Medical Centre Amsterdam Public Health Amsterdam The Netherlands; ^24^ Wilhelmina Children’s Hospital Utrecht The Netherlands; ^25^ University of Twente, Enschede, The Netherlands, and International Consortium for Health Outcomes Measurement London UK

## Abstract

**Objective:**

The implementation of value‐based health care in inflammatory arthritis requires a standardized set of modifiable outcomes and risk‐adjustment variables that is feasible to implement worldwide.

**Methods:**

The International Consortium for Health Outcomes Measurement (ICHOM) assembled a multidisciplinary working group that consisted of 24 experts from 6 continents, including 6 patient representatives, to develop a standard set of outcomes for inflammatory arthritis. The process followed a structured approach, using a modified Delphi process to reach consensus on the following decision areas: conditions covered by the set, outcome domains, outcome measures, and risk‐adjustment variables. Consensus in areas 2 to 4 were supported by systematic literature reviews and consultation of experts.

**Results:**

The ICHOM Inflammatory Arthritis Standard Set covers patients with rheumatoid arthritis (RA), axial spondyloarthritis, psoriatic arthritis, and juvenile idiopathic arthritis (JIA). We recommend that outcomes regarding pain, fatigue, activity limitations, overall physical and mental health impact, work/school/housework ability and productivity, disease activity, and serious adverse events be collected at least annually. Validated measures for patient‐reported outcomes were endorsed and linked to common reporting metrics. Age, sex at birth, education level, smoking status, comorbidities, time since diagnosis, and rheumatoid factor and anti‐citrullinated protein antibody lab testing for RA and JIA should be collected as risk‐adjustment variables.

**Conclusion:**

We present the ICHOM inflammatory arthritis Standard Set of outcomes, which enables health care providers to implement the value‐based health care framework and compare outcomes that are important to patients with inflammatory arthritis.

## Introduction

The inflammatory arthritides are a group of systemic, immune‐mediated rheumatic conditions, characterized by synovitis or inflammation of periarticular tissues and joint damage. The lifetime risk of adult‐onset inflammatory arthritis has been estimated to be ~6% of the US population 1. The availability of strategies to diagnose the diseases earlier, and the availability of biologic and targeted small molecule therapies, in combination with early, tightly controlled treatment strategies have led to relevant improvements in outcomes for many patients over the last decades 2, 3. However, these improvements have also resulted in an increased financial burden on health care systems 4, 5.

**Significance & Innovations**

- Standards for measuring and comparing treatment outcomes that matter to patients with inflammatory arthritis that are globally implementable are currently lacking.
- We used a modified Delphi procedure and systematic reviews of the literature to develop a standard set of outcomes that matter to patient with inflammatory arthritis.
- The patient‐reported outcome measures we recommend for measuring pain, activity limitations, fatigue and assessment of overall emotional and physical health impact were linked to a common item response theory–based common metric, so that users of the set can select their preferred instrument for measuring these outcomes.

The prevalence and case recognition of inflammatory arthritis is expected to increase further over the next decade, particularly in less economically developed countries 6. Hence, it will be increasingly important to optimize care and allocate available resources efficiently to improve or maintain quality of care. Value Based Healthcare (VBHC) is a framework for improving the quality and efficiency of health care, in which improving value for the patient is the central concept 7. Value is defined as the patient outcomes achieved, relative to financial costs for obtaining those outcomes. Within this framework, value can be increased by improving patient outcomes or by delivering the same outcomes at a lower cost. Public reporting of patient outcomes by health care providers is proposed as a mechanism that will accelerate identification and adoption of high value care, through shared learning and promoting benchmarking of outcomes that matter to patients.

In order for outcomes to be comparable between different health care providers, exact definitions for each relevant outcome are required. The outcomes need to be feasible to be collected in a variety of health care systems, and a set of relevant risk‐adjustment variables should be included to ensure risk‐adjusted comparisons of outcomes between providers that serve different patient populations. The International Consortium for Health Outcomes Measurement (ICHOM) initiative is working toward the global implementation of VBHC by developing standard sets of patient outcomes for a range of medical conditions 8. These standards are intended to be implemented in routine clinical practice and therefore complement earlier core sets and reporting standards intended for clinical research, including the work of the Outcome Measures in Rheumatology group 9.

The ICHOM process is grounded in a conceptual framework which distinguishes 3 hierarchically ordered tiers of outcome, including health status achieved/retained, the process of recovery, and the sustainability of health 10. In order to select the most relevant outcomes, outcome measures, and risk adjustment variables for particular conditions, various stakeholders including patients, physicians, policymakers and outcome experts are engaged in a consensus‐building process that is supported by a systematic evaluation of the available evidence base, including critical evaluation of available instruments and evidence supporting their measurement properties. In order to further encourage the adoption and implementation of VBHC in rheumatology, the aim of our study was to develop a globally applicable set of outcome measures that reflect outcomes that matter to patients with inflammatory arthritis, for providers to track in their clinical practice.

## Patients and Methods

#### Working group

A working group of outcome experts (n = 24) was convened by ICHOM. Working group members were carefully selected to ensure representation of relevant professional disciplines, different geographic areas, and the patient perspective. The working group included patient representatives (n = 6), registry leaders, and members with a professional background in adult and pediatric rheumatology, nursing, epidemiology, psychology, rehabilitation medicine, physiotherapy, and psychometrics. Working group members of all 6 inhabited continents were included. The efforts of the working group were guided and facilitated by a core project team.

#### Working group process

A modified Delphi approach that has been developed by ICHOM and was previously applied by ICHOM to develop standards for a number of other conditions 9, 11, 12, 13, 14, 15, 16, 17, 18, 19, 20, 21, 22, 23, 24, 25, 26 was used. The process involved reaching consensus in 4 major decision areas: determination of which inflammatory arthritis conditions and treatments to include in the Standard Set, a minimally sufficient set of outcomes relevant for each of the conditions, standardized definitions and time points for assessing these outcomes, and standardized definitions for risk‐adjustment variables to ensure fair comparisons between health care providers who wish to implement the set. Each decision area was discussed during a dedicated video conference. A list of potentially relevant items (i.e., conditions/domains/time points/risk‐adjustment variables) to be included in the Standard Set, along with supporting evidence (see below), was prepared by the project team preceding each meeting. These items were identified in a series of systematic literature reviews and/or consultation with external experts on the topic under consideration. A summary of the preparatory work was provided to working group members preceding each video conference. During each meeting, the items were discussed and expanded on or revised based on the input of the working group. Following each video conference, the project team circulated a summary of the discussions and a survey that asked working group members to provide feedback and vote on each item considered for inclusion in the Standard Set. For voting during the final survey, a 9‐point Likert scale ranging from 1 (“not recommended”) to 9 (“essential to have”) was used. Items were included in the Standard Set if they were rated ≥7 by at least 70% of the working group or excluded if rated ≤3 by at least 70% of the working group. In other cases, the result was considered inconclusive and the item was discussed again during the following video conference. The conduct and reporting of the Delphi process followed reporting guidelines for core outcome set development using the Delphi approach (see Supplementary Table 1, available on the *Arthritis Care & Research* web site at http://onlinelibrary.wiley.com/doi/10.1002/acr.23799/abstract) 27.

#### Preselection of relevant patient outcome domains

Preceding the video conference on the selection of outcome domains, 2 separate systematic literature reviews were performed in December 2016 using the PubMed database, in order to identify outcomes relevant to patients for the included conditions that were modifiable in principle and feasible to implement. In the first search, we used the “Cochrane Highly Sensitive Search Strategy for identifying randomized trials” 28 to identify 25 recent randomized trials in each of the conditions included in the Standard Set. For each randomized trial, we checked the relevant online repositories and conducted a second PubMed search using the name of each trial and trial registration number, in order to find additional publications on the same study. All outcomes assessed in each trial were extracted. Reports on randomized trials in languages other than English were excluded. In the second search, we identified reports on qualitative studies in which patients with 1 of the relevant conditions were asked about the most important outcomes of their disease. We included only papers in which an open‐ended question format was used, in order to prevent investigator‐imposed biases on the list of patient‐generated outcome domains. All outcome domains considered important by patients were extracted from each paper by 2 reviewers independently. Disagreements were resolved during a consensus meeting with a third reviewer present. Previously published core set recommendations for outcome measurement in randomized trials were also consulted, as was the European League Against Rheumatism standardized data set for observational research 29, 30, 31, 32, 33. Finally, 2 patient advisory group sessions with inflammatory arthritis patients from the Netherlands and the US were organized by the project team to serve as a check on the comprehensiveness of the list of identified patient outcomes. The patient advisory group protocol was exempt from ethical review by the Chesapeake Institutional Review Board.

#### Preselection of outcome measures

All outcome measures used in any randomized trial identified in the initial systematic review, recommended for inclusion by working group members or previously endorsed by relevant consensus statements, were considered for inclusion. The instruments were reviewed with respect to outcome domains, evidence supporting psychometric properties, feasibility, licensing fees, and degree to which they were established in the field. In order to support this process, a systematic literature review was performed in May 2017 to identify papers that had reported on the measurement properties of 26 potentially relevant patient‐reported outcome measures (PROMs). The methodologic quality of the 159 identified papers was assessed using the Consensus‐Based Standards for the Selection of Health Status Measurement Instruments (COSMIN) checklist 34. The studies that were of high methodologic quality were then used to rate the measurement properties of the 26 PROMs, using quality criteria proposed by Terwee et al and the International Society for Quality of Life Research 35, 36. Comprehensibility, cost, and time needed for completion were all assessed to determine the feasibility of implementing specific PROMs. The Flesch‐Kincaid grade level was calculated for the English language version of each PROM 37, information about fees payable for use of the instrument was retrieved from the copyright owner's website when applicable, and information on time to complete was retrieved from a previous series of reviews 38.

#### Preselection of risk‐adjustment variables

A preliminary list of risk‐adjustment variables was extracted from published reviews on risk factors and validated risk models. Previously published ICHOM Standard Sets were reviewed for definitions of demographic and socioeconomic variables.

#### External validation by health professionals and patient experts

After proceeding through all of the Delphi rounds, the preliminary Standard Set was made available and sent to various stakeholders for review (http://www.ichom.org). A patient survey was distributed through national patient organizations and the networks of the project team and working group members. Patients were asked to rate the importance of each outcome using a 9‐point Likert scale ranging from “1 = not at all relevant” to “9 = essential,” and were given the opportunity to suggest additional outcomes. Health professionals were recruited via the professional networks of the working group members and project team. Health professionals were asked to rate the relevance of each domain, provide feedback on feasibility of implementation of the Standard Set in clinical practice, and rate the appropriateness of the risk‐adjustment variables and time points for assessment.

## Results

#### Scope

At the start, it was recognized by the working group that the same treatment goals and longitudinal outcomes (pain, physical function, fatigue) are relevant to most types of inflammatory arthritis. The ICHOM Inflammatory Arthritis Standard Set was therefore designed to evaluate treatment outcomes of patients with rheumatoid arthritis (RA), axial spondyloarthritis (SpA) and psoriatic arthritis (PsA), as well as juvenile idiopathic arthritis (JIA), and applies to all treatments for these conditions, including medication, surgery, and physical and occupational therapies. All working group members voted to include RA, axial SpA, and PsA, and 82% voted to include JIA. The inclusion of gout (59% voted to include initially), connective tissue diseases (41%) and vasculitis (36%) was also considered, especially as few outcome recommendations are available for the latter 2 conditions. However, after revisiting this topic at a subsequent meeting, it was decided that their inclusion might lead to a generic set of outcomes, which might insufficiently capture the outcomes that matter to patients with individual conditions, due to the variety of disease manifestations and disease courses.

#### Outcome domains

Twenty‐four outcome domains were initially identified in the 130 randomized trial reports and 28 qualitative studies that were identified in the systematic literature reviews (references available upon request from the corresponding author). This list was expanded upon and refined several times based on group discussions with working group members. The final consolidated list of outcomes is presented in Table 1, together with a summary of both systematic reviews and the final voting results. The list of outcome domains assessed in randomized trials and their rank ordering reflects a preference in trials for clinical measures of disease manifestations and patient‐reported outcomes of symptoms and their direct impact on functioning. The list and rank ordering of patient‐generated outcome domains, on the other hand, somewhat deemphasized the importance of outcome measures that reflect the pathophysiology of the specific disease and included a wider variety of outcomes that reflect the different ways arthritis impacts the daily lives of patients. Besides PROMs of symptoms and basic functioning, the patient‐generated list also included more generic outcomes, such as overall psychological well‐being and participation restrictions. To characterize the core symptoms and their direct effect on functioning from the patient perspective, the working group recommends that providers assess pain, fatigue, and activity limitations (i.e., physical function). These were the most frequently used outcome domains in randomized trials and were reported as important by patients in almost all of the reviewed qualitative studies. These outcome domains were also the most frequently endorsed domains in the individual core sets for randomized trials of the respective conditions. In order to assess the impact of inflammatory arthritis on the daily lives of patients more broadly, the working group recommends an assessment of overall emotional and physical health impact, and work/school/housework productivity. Participation restrictions other than work or school productivity were also considered important. However, this domain was eventually excluded because of significant overlap with other included domains, and because experience with available measurement instruments is currently limited.

**Table 1:** Final list of outcomes considered for inclusion in ICHOM IA set^a^

Outcome	Qualitative studies in which outcome was reported as important disease outcome	No. of IA conditions for which outcome is included in clinical trial core set	No. of IA conditions for which outcome was measured in ≥1 clinical trial, but not in core set	Working group members voted for inclusion (%)
Pain	27 (96)	3	1	100
Physical function	26 (93)	4	NA	100
Adverse events	11 (39)	0	4	95
Fatigue	23 (82)	2	2	90
Work/school ability and productivity	14 (50)	0	3	90
Overall physical and mental health impact	NA	4	NA	86
Inflammatory disease activity	6 (21)	4	NA	84
Participation restrictions	18 (64)	0	0	55
Joint damage	7 (25)	2	1	45
Mortality	1 (4)	0	0	40
Psychological well‐being	22 (79)	0	0	35
Sleep	10 (36)	0	2	35
Coping & self‐management	16 (57)	0	0	33
Financial impact	3 (11)	0	0	25
Joint stiffness	15 (54)	0	4	20
Joint range of motion	0 (0)	1	0	15
Physical appearance	7 (25)	0	0	10
Sexual functioning	8 (29)	0	0	10
Cognitive functioning	5 (18)	0	0	5
Fever	0 (0)	0	1	0

a Values are the number (%) unless indicated otherwise. ICHOM = The International Consortium for Health Outcome Measurement; IA = inflammatory arthritis; NA = not applicable.

Assessments of inflammatory disease activity and therapeutic response are further recommended as measures of disease control, because the absence of signs and symptoms of disease is the primary treatment target for inflammatory arthritis and disease activity was considered the main determinant of overall impact of disease. Finally, adverse events should be recorded as a measure of disutility of care.

#### Outcome measures

The list of recommended PROMs is shown in Table 2. Supplementary Appendix 1 provides an overview of characteristics and ratings assigned to the measurement properties of these PROMs and includes an overview of the criteria used for assigning ratings (available online on the *Arthritis Care & Research* web site at http://onlinelibrary.wiley.com/doi/10.1002/acr.23799/abstract).

**Table 2:** Overview of measures endorsed for assessing patient‐reported outcomes included in ICHOM IA Standard Set^a^

Outcome, endorsed instruments	Construct validity	Reliability	Responsiveness	Flesch‐Kincaid grade level^b^
Pain				
NRS/VAS	>1	>1	>1	7
SF‐36 bodily pain	>1	>1	>1	8
PROMIS Short	0	0	1	6
Form v1.0– Pain				
Interference 8a				
PedsQL aches and pain^c^	0	1	0	0
Fatigue				
BRAF‐MD	1	>1	1	5
FACIT‐F	1	>1	>1	3
NRS/VAS	>1	1	>1	8
PROMIS Short Form	0	0	1^d^	5
v1.0– Fatigue 8a				
PedsQL 4.0 fatigue^c^	0	1	0	0
Activity limitations				
HAQ DI	>1	>1	>1	3
HAQ‐II	1	>1	0	4
MHAQ	1	>1	>1	3
PROMIS Short Form	1	0	e	4
v2.0 – Physical				
Function 10a				
MDHAQ score	1	>1	1	3
BASFI	0	>1	>1	7
C‐HAQ	1	>1	>1	4
JAMAR	0	0	0	2
Health impact				
PROMIS global health	0	0	0	8
EQ‐5D^f^	>1^g^	>1	>1	6
SF‐6D	1	>1	>1	9
RAID (for RA)	0	>1	1	10
PSAID (for PsA)	0	>1	1	12
ASAS Health Index	1	>1	0	6
Patient/parent global assessment (NRS or VAS)	0	>1	>1	7
Work/school/ housework ability and productivity, WPAI	1	1	1	8

a Values are the number of studies of good methodologic quality that noted favorable properties, unless indicated otherwise. ICHOM = International Consortium for Health Outcome Measurement; IA = inflammatory arthritis; NRS = numerical rating scale; VAS = visual analog scale; SF‐36 = Short Form 36 Health Survey; PROMIS = Patient‐Reported Outcome Measurement Information System; PedsQL = Pediatric Quality of Life Inventory; BRAF‐MD = Bristol Rheumatoid Arthritis Fatigue Multidimensional Questionnaire; FACIT‐F = Functional Assessment of Chronic Illness Therapy‐Fatigue; HAQ DI; Health Assessment Questionnaire Disability Index; MHAQ = Modified Health Assessment Questionnaire Disability Index; MDHAQ = Multidimensional Health Assessment Questionnaire; BASFI = Bath Ankylosing Spondylitis Functional Activity Index; C‐HAQ = Childhood Health Assessment Questionnaire; JAMAR = The Juvenile Arthritis Multidimensional Assessment Report; EQ‐5D = EuroQol 5 dimensions; SF‐6D = Medical Outcome Study Short Form 6D; RAID = Rheumatoid Arthritis Impact of Disease; RA = rheumatoid arthritis; PSAID = Psoriatic Arthritis Impact of Disease; PsA = psoriatic arthritis; ASAS = Assessment of SpondyloArthritis international Society; WPAI =Work Productivity and Activity Impairment Questionnaire.

b Estimated using the Flesch‐Kincaid grade level statistic.

c Instrument is intended for pediatric populations.

d Unfavorable properties according to 1 study with good methodologic quality.

e Mixed findings in studies of good methodologic quality.

f Including EQ‐5D‐Y for pediatric patients.

g Unfavorable properties according >1 study of good methodologic quality.

The working group agreed that a key property was feasibility in different settings globally. Therefore, instruments with a large number of items, or that could not be hand‐scored were avoided. All endorsed PROMs are available in multiple languages and for each outcome domain, at least 1 PROM is recommended that was judged to have sufficient evidence supporting its measurement properties, based on the results of the systematic review. On the other hand, some instruments were included that do not (yet) meet psychometric standards of the COSMIN checklist. Several Patient‐Reported Outcomes Measurement Information System (PROMIS) measures were included so that experience with innovative Item Response Theory (IRT)–based measures could accumulate. The RA and PsA impact of disease scores and the Assessment of SpondyloArthritis international Society (ASAS Health index) were recommended because these are patient/International Classification of Functioning, Disability, and Health–derived composite scores that assess the important domains of impact of RA and PSA, and axial SpA, respectively. An overview of the various measures that are recommended by the clinical guidelines issued by various national and international rheumatology societies is provided (see Supplementary Table 2, available on the *Arthritis Care & Research* web site at http://onlinelibrary.wiley.com/doi/10.1002/acr.23799/abstract. For each of the ICHOM outcome domains, the endorsed outcome measures are congruent with the various guidelines. Users of the Standard Set may select preferred instruments to assess each outcome from the list of endorsed PROMs presented in Table 2. The shortest recommended combination of PROMs to assess all outcome domains is the numerical rating scale/visual analog scale (VAS) to measure fatigue, overall emotional and physical health impact, and pain; the Work Productivity and Activity Impairment questionnaire to measure work/school/housework ability and productivity; and the Health Assessment Questionnaire II to measure activity limitations. This combination of PROMs is free to use for all users and totals 19 questions, which most patients should be able to fill out in 5 minutes. The endorsed PROMs of pain, fatigue, overall well‐being, and activity limitations have been linked to a common reporting metric, such that outcomes can be compared between providers that have used different instruments 39. Linked scores for benchmarking outcomes using any of the recommended instruments of the Standard Set can be obtained from http://www.tihealthcare.nl/en/expertise/common-metrics.

In order to track disease activity and therapeutic response, it is proposed that major evidence‐based guidelines are followed 40, 41. Patients and health care providers should specify target disease activity levels for individual patients (preferably remission; if not, feasible low disease activity) and assess at each visit whether this target was achieved. Disease activity should be monitored using a validated and internationally recognized clinical composite score.

#### Risk‐adjustment variables

Most risk‐adjustment variables included in the set (Table 3) apply to all patients, and care was taken to include risk‐adjustment variables that are relevant and applicable in a variety of health care systems. Year of birth and sex were included as demographic variables. Education level was ultimately chosen as the only indirect measure of social economic status (SES). Other SES‐related variables were considered important, but difficult to collect, due to restrictions on recording race/ethnicity in some countries, area‐based measure of SES possibly being unavailable for each country, and patients potentially feeling reluctant to report on their income/wealth. For baseline status indicators, we included smoking status, comorbidities, diagnosis, time since diagnosis, and rheumatoid factor and anti–cyclic citrullinated protein antibody for RA and JIA. HLA–B27 was excluded since it is not routinely collected in the health care system. Comorbidities should be assessed using the Rheumatic Disease Comorbidities Index 42, modified to include central sensitization to pain (e.g., fibromyalgia) and obesity. In order to avoid misclassification of early symptoms that may or may not reflect those specific to the inflammatory arthritis diagnosis of interest, we elected to include time since diagnosis rather than time since symptom onset.

**Table 3:** Case‐mix variables^a^

Variable	Definition (response options)	Timing^b^	Data source
Age	Year of birth	Baseline	Patient
Sex	Sex at birth (Female/male)	Baseline	Patient
Smoking status	Never/former /current	Baseline	Patient
Education level	Highest attained education ISED classification (none/primary/secondary/tertiary)	Baseline	Patient
Comorbidities	Present/absent/unknown: chronic lung disease, myocardial infarction, other heart disease, stroke, hypertension, diabetes mellitus, fracture, depression, ulcer or stomach problem, cancer, central sensitization to pain, obesity (i.e., BMI ≥30)	Baseline	Clinical
Diagnosis	Physician reported diagnosis (RA/SpA/PsA/JIA)	Baseline and annually	Clinical
Disease duration	Year of diagnosis	Baseline	Clinical
Immunologic^c^	Rheumatoid factor and ACPA positivity (yes/no)	Baseline	Clinical

a ISED = Institute for the Study of Education and Human Development; BMI = body mass index; RA = rheumatoid arthritis; SpA = spondyloarthritis; PsA = psoriatic arthritis; JIA = juvenile idiopathic arthritis; ACPA = anti‐citrullinated protein antibody.

b Baseline defined as first measurement for patient.

c Only for RA and JIA.

#### Data collection time points

To allow meaningful outcomes comparisons between health care providers, we recommend that all risk‐adjustment variables to be collected at the first assessment. All PROMs and clinical measures should also be collected at the first assessment and annually thereafter. In instances of active disease, we recommend that the patient's disease activity status be recorded at least every 6 months, but likely more frequently, at the discretion of the patient and their health care provider. Adverse events should be collected at each assessment point after baseline. A reference guide with detailed instructions for implementation and exact definitions for all of the data elements can be downloaded (http://www.ICHOM.org). Finally, we stress that these recommendations are intended only for quality improvement purposes and should not be understood as more than minimally acceptable clinical guidelines in patients with established disease. Especially in patients with early disease, more frequent monitoring may be required.

#### Open review

**Figure 1:**
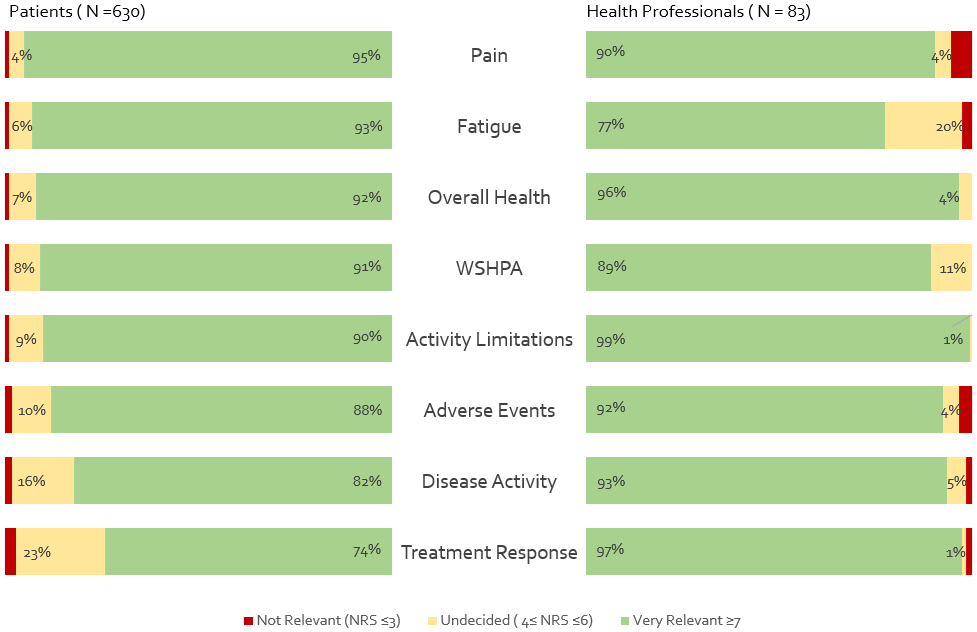
Relevance of outcomes included in The International Consortium for Health Outcome Measurement inflammatory arthritis set according to patient and health professional open review. WSPHA = Work/School/Household productivity and ability; NRS = numerical rating scale.

Eighty‐three health care professionals, the majority of which (95%) were clinician/researchers, and 630 people living with inflammatory arthritis from the US, France, Argentina, The Netherlands, and Brazil reviewed the Standard Set. All outcomes included in the ICHOM Inflammatory Arthritis Set were considered very relevant by patients and health care professionals (Figure 1). Similar to the results of the systematic reviews that were used for identifying outcome domains, patients considered clinical measures slightly less relevant compared with the patient‐reported outcomes. A large majority of patients (81.3%) felt that the set comprehensively covers all the relevant outcome domains of their disease. The health care professionals predominantly shared this view. Only 3 outcomes were suggested to be missing by >1 reviewer: financial impact (n = 2), joint damage (n = 2), and patient satisfaction (n = 3). Psychological well‐being (12.6%) and participation restrictions (5.4%) were the only outcomes that were reported as missing from the set by >2% of patient reviewers (see Supplementary Appendix 1, available on the *Arthritis Care & Research* web site at http://onlinelibrary.wiley.com/doi/10.1002/acr.23799/abstract). The included risk‐adjustment variables were rated very relevant by 91.8% of health professionals.

## Discussion

We present a standard set of outcomes for inflammatory arthritis that health care providers worldwide can use in routine clinical care to help quantify the value provided for patients in different centers, countries, and health care systems. This Standard Set was developed through consensus of an international working group with expertise across a range of disciplines relevant to outcome assessment and care for patients with inflammatory arthritis. We used a multiple methods approach, in which extensive patient input as well as published qualitative and quantitative data were used to develop a minimally sufficient set of outcomes that we believe represents outcomes that matter to patients with inflammatory arthritis. We also proposed time points for data collection and relevant risk‐adjustment variables to enable comparisons between providers with different patient populations. Feasibility of implementation in different health care systems was a central priority. Therefore, we included PROMs that are not only widely accepted measures of the respective domains but that are also available in multiple translations, and for each outcome there is at least 1 PROM free to use. However, for the use of several instruments, including the Pediatric Quality of Life Inventory, the Medical Outcome Study Short Form 6D, Functional Assessment of Chronic Illness Therapy (FACIT), and EuroQol 5‐domain license fees may apply. Patient‐Reported Outcomes Measurement Information System (PROMIS) has also introduced financial charges for the use of some of their products, including their computerized adaptive tests. Using the PROMIS Assessment Centre platform will incur a $5,000 USD charge per study per year.

One of the challenges faced with international standardization of patient outcomes data collection is that a variety of well validated and frequently used PROMs are typically available to assess the different patient outcomes. The working group for the ICHOM Depression & Anxiety and Chronic Kidney Disease Standard Sets previously responded to this challenge by endorsing PROMs that can be mapped to the PROMIS metric, using resources provided by the PROMIS PROsetta project 43, 44. This way, users of these sets use 1 PROM for each domain, but results can be scored on the PROMIS metric. In the work on the ICHOM inflammatory arthritis Standard Set presented here, this is taken one step further, by linking multiple PROMs to an IRT‐based common reporting metric 45. This makes it easier for new or ongoing data collection initiatives to contribute their data, since it allows users of the ICHOM inflammatory arthritis set to choose 1 instrument from a number of alternatives for each domain. Provided that 1 of the endorsed instruments (Table 2) is collected, outcomes can be compared with those from other health care providers who use the ICHOM Standard Set. For example, outcomes assessed using the VAS scale for fatigue can be directly compared with outcomes of a different group of patients assessed using the FACIT–Fatigue subscale. In principle, PROMs could be added to and removed from the list of endorsed instruments, without affecting comparability of the outcomes. The ICHOM list of recommended PROMs overlaps significantly with current clinical guidelines. Moreover, the results of 2 systematic reviews of various national RA patients’ registries show that the majority of the PROMs that are currently collected in the reviewed registries are also included in the ICHOM Inflammatory Arthritis Standard Set. The IRT approach also allows each of the ICHOM inflammatory arthritis outcomes to be assessed using computerized adaptive tests, which would help achieve optimally precise scores with minimal numbers of questions 45, 46.

Since the ICHOM Inflammatory Arthritis Standard Set is intended to reflect outcomes that are important to patients, the extensive input from patients is a strength of this work. We included 6 patient representatives in the working group, derived the list of outcomes from published qualitative studies in which patients reported outcomes that matter to them, organized 2 patient advisory group sessions with patients that were not included in the working group to review the final list of outcomes to be voted on by working group members, and the final version of the Standard Set was reviewed by 630 patients from various countries. The inclusion of working group members with diverse geographic and professional backgrounds is also a strength.

We do, however, acknowledge that different results might have been obtained had other working group members been selected. We also realize that it may prove challenging to collect all the requested information for all health care providers at all time points. In particular, inflammatory disease activity may prove logistically challenging to track in some health care systems, as it requires clinical assessment of joint involvement and, in some cases, laboratory assessments. In such situations, we would encourage users of the set to at least monitor the PROMs. All patient‐reported outcomes can be collected using a minimum of 20 items, which could be further reduced using computerized adaptive testing or targeted short forms. Finally, we acknowledge that the value of the ICHOM Inflammatory Arthritis Standard Set has not yet been proven in practice. ICHOM aims to partner with several interested institutions to pilot test the Standard Set. Furthermore, a steering committee has been established that will periodically review the Standard Set, including lessons learned from the pilot phase and other applications of the set. This will include, but will not be limited to, reviewing PROMs that are endorsed in the Inflammatory Arthritis Standard Set, the ease in accessing and monitoring these PROMs, and the outcomes related to personal goals that individual patients identify.

In summary, we propose a standard set of outcomes for patients with inflammatory arthritis that providers of care for patients with inflammatory arthritis can track to facilitate the global reporting of outcome data and shared learning. A detailed reference guide is available (http://www.ichom.org).

## Author Contributions

All authors were involved in drafting the article or revising it critically for important intellectual content, and all authors approved the final version to be submitted for publication. Dr. Oude Voshaar had full access to all of the data in the study and takes responsibility for the integrity of the data and the accuracy of the data analysis.

### Study conception and design

Oude Voshaar, Das Gupta, van de Laar, Vonkeman.

### Acquisition of data

Oude Voshaar, Das Gupta, Bijlsma, Boonen, Chau, Courvoisier, Curtis, Ellis, Ernestam, Gossec, Hale, Hornjeff, Leung, Lidar, Mease, Michaud, Mody, Ndosi, Opava, Pinheiro, Salt, Soriano, Taylor, Voshaar, Weel, de Wit, Wulffraat, van de Laar, Vonkeman.

### Analysis and interpretation of data

Oude Voshaar, Das Gupta, Bijlsma, Boonen, Chau, Courvoisier, Curtis, Ellis, Ernestam, Gossec, Hale, Hornjeff, Leung, Lidar, Mease, Michaud, Mody, Ndosi, Opava, Pinheiro, Salt, Soriano, Taylor, Voshaar, Weel, de Wit, Wulffraat, van de Laar, Vonkeman.
