# Supplementary material for: International Consortium for Health Outcome Measurement Set of Outcomes That Matter to People Living With Inflammatory Arthritis: Consensus From an International Working Group
Source: Arthritis Care Res (Hoboken). 2019 Nov 29;71(12):1556–65. doi: 10.1002/acr.23799 (PMC6900179; doi:10.1002/acr.23799)
Supplement: Supplementary file 1 [file ACR-71-1556-s001.docx]

| **Checklist item** | **Results** |
| --- | --- |
| **Size and Composition of the Panel** |  |
| The total number of participants invited | 29 (3 declined to participate, 1 agreed to participate but never participated) |
| The number of participants who completed the first round | 24 (1 WG member dropped out after the first round of voting) |
| **Methodology of the Delphi Process** |  |
| Mode of survey administration | E-mail survey |
| Information about outcomes provided preceding votes | Described in methods section |
| how were outcomes to be voted on initially identified | systematic literature review as described in methods |
| What was asked in subsequent rounds | Participants were asked to rate the relevance of each item on the list and to provide comments. Forms used are available on request from the corresponding author |
| Feedback to participants after each round | The voting results were fed back to the participants between rounds, together with all comments provided by participants |
| Level of anonymity | Votes remained anonymous |
| Definition of consensus | Described in the methods section |
| Were non-responders invited to subsequent rounds | Non responders were not excluded |
| **Results** |  |
| Number of participants invited to each round | all participants were invited to vote in each round |
| Number who completed every round | median (min- max) survey completion proportion was 0.88 (0.83 - 0.95) |
| Results for each outcome scored by participants in each round | Available on request from the corresponding author |
| Measure of group response for each outcome scored by participants in the final round | Available on request from the corresponding author |
| Distribution of response for each outcome scored by participants in the final round | Available on request from the corresponding author |
| A comprehensive list of all the outcomes that participants agreed should be included in the core set | Described in the results section |
