# Supplementary material for: International Consortium for Health Outcome Measurement Set of Outcomes That Matter to People Living With Inflammatory Arthritis: Consensus From an International Working Group
Source: Arthritis Care Res (Hoboken). 2019 Nov 29;71(12):1556–65. doi: 10.1002/acr.23799 (PMC6900179; doi:10.1002/acr.23799)
Supplement: Supplementary file 2 [file ACR-71-1556-s002.docx]

| Institution | condition | Fatigue | Pain | Physician global | patient global | physical function | Stiffness | HRQOL | Sleep disturbance | Mental Health |
| --- | --- | --- | --- | --- | --- | --- | --- | --- | --- | --- |
| French Society for Rheumatology | RA |  |  |  |  | HAQ |  |  |  |  |
| EUlAR/ Assessment of Spondyloarthritis international Society | AS | NRS | NRS/VAS |  | NRS/VAS | BASFI | NRS/VAS |  |  |  |
| RODS | RA |  | VAS/NRS | VAS/NRS | NRS/VAS | HAQ |  | EQ5D |  |  |
| Brazilian Society of Rheumatology | RA |  |  |  |  | mHAQ or HAQ-DI, |  |  |  |  |
| Hong Kong Society of Rheumatology | RA |  |  |  |  | HAQ pf10 |  |  |  |  |
| PANLAR | RA | FACIT | VAS/NRS | VAS/NRS | VAS/NRS |  |  | sf-36, HUI |  |  |
| The Spanish Society of Rheumatology | RA |  | VAS/NRS | VAS/NRS | VAS/NRS | HAQ |  |  |  |  |
| the Mexican college of rheumatology | RA |  |  |  |  |  |  |  |  |  |
| Dutch Society for Rheumatology | RA |  |  |  |  | HAQ |  |  |  |  |
| Dutch Society for Rheumatology | AS |  |  |  |  | BASFI |  |  |  |  |
| Brazilian Society of Rheumatology | Psa |  |  |  |  |  |  |  |  |  |
| Brazilian Society of Rheumatology | As |  |  |  |  |  |  |  |  |  |
| The National Institute for Health and Care Excellence (UK) | RA |  |  |  |  | HAQ |  |  |  |  |
| The National Institute for Health and Care Excellence (UK) | AS |  | VAS |  |  |  |  |  |  |  |
| European League Against Rheumatism | RA |  |  |  | NRS |  |  |  |  |  |
| European League Against Rheumatism | Psa |  |  |  |  |  |  |  |  |  |
| American College for Rheumatology | RA |  |  |  |  | HAQ, MDHAQ, PROMIS, HAQ-II |  |  |  |  |
| American College for Rheumatology | AS | NRS, Fatigue questionnaires | NRS, Sf-36 |  |  | BASFI, Sf-356 Pf10 | NRS | sf-36, ASQOL | NRS | SF-36 |
| American College for Rheumatology | JIA |  |  |  |  |  |  |  |  |  |
| American College for Rheumatology | Psa |  |  |  |  |  |  |  |  |  |
| British Society for Rheumatology & British Health Professionals in Rheumatology | AS |  | VAS |  | VAS | BASFI |  |  |  |  |
| British Society for Rheumatology & British Health Professionals in Rheumatology | Psa |  |  |  |  |  |  |  |  |  |
| French Society for Rheumatology | AS |  |  |  |  |  |  |  |  |  |
