# Supplementary material for: International Consortium for Health Outcome Measurement Set of Outcomes That Matter to People Living With Inflammatory Arthritis: Consensus From an International Working Group
Source: Arthritis Care Res (Hoboken). 2019 Nov 29;71(12):1556–65. doi: 10.1002/acr.23799 (PMC6900179; doi:10.1002/acr.23799)
Supplement: Supplementary file 3 [file ACR-71-1556-s003.pdf]

Outcome measures were characterized by following criteria:

- Reliability
  - Internal consistency
  - Test-retest reliability
- Validity
  - Construct validity
- Responsiveness
- Alternate versions agreement
- IRT

	Method	Rating	Criteria (based ond Terwee et al. 2007)[2]
Reliability	Internal consistency	?	No information found on internal consistency or not all information for '+' reported (e.g. no factor analyses or comparable) OR doubtful design or method
		+	At least limited evidence for unidimensionality or positive structural validity AND Cronbach's alpha(s) or equivalent ≥ 0.70
		-	Criteria for '+' not met
	Test-retest reliability	?	No information found on reliability OR ICC or weighted Kappa not reported OR doubtful design or method
		+	ICC or weighted Kappa ≥ 0.70
		-	Criteria for '+' not met
Validity	Construct validity	?	No information found on construct validity or doubtful design or method (e.g. no hypotheses)
		+	Specific hypotheses were formulated AND at least 75% of the results are in accordance with the hypotheses
		-	Criteria for '+' not met
Responsiveness	Responsiveness	?	No information found on responsiveness or doubtful methods (e.g. no rationale for score change)
		+	For internal responsiveness: longitudinal study design and therapy with established efficacy, for external responsiveness: appropriate external criterion AND standardized change scores of at least small magnitude ES = ≥0.20 in case of hypothesized improvement/deterioration and standardized change scores <0.20 if scores are hypothesized to remain stable.
		-	Criteria for '+' not met

Copyright © 2017 by the International Consortium for Health Outcomes Measurement. All rights reserved.

PAIN			
	Name	VAS	NRS
GENERIC INFO	Full name	Visual analogue scale	Numerical rating scale
	Description	A continuous scale, usually presented as a 100 mm horizontal line, anchored by two verbal descriptors, representing opposing extremes of the measured health state Respondents are asked to mark the location along this line that best represents their level of the measured health state	A numerical rating scale in which a patient selects the whole number (0–10 integers) that best reflects the intensity of the measured trait
	# of items	1	1
	Scale type	Most commonly anchored by "no pain" (score of 0) and "pain as bad as it could be" or "worst imaginable pain" (score of 100 [100-mm scale])	An 11-point numeric scale (NRS 11) with 0 representing one pain extreme (e.g., "no pain") and 10 representing the other pain extreme (e.g., "pain as bad as you can imagine" and "worst pain imaginable")
	Recall period	Varies, but most commonly 1 day	Varies, but most commonly 1 day
	Scoring	Using a ruler, the score is determined by measuring the distance (mm) on the 10-cm line between the "no pain" anchor and the patient's mark, providing a range of scores from 0–100	The number that the respondent indicates on the scale to rate their pain intensity is recorded. Scores range from 0–10.
**RELIABLITY**	Test-retest	0.77 (0.70 - 0.87) N = 3	?
	Internal consistency	n/a	n/a
**VALIDITY**	Construct validity	100% N = 2	100% N = 1
**RESPONSIVENESS**	(Ability to detect change)	100% N = 8	100% N = 5
**IRT**		n/a	n/a
**Paper/electronic agreement (ICC)**		0.92 (0.87 – 0.99) N = 3	0.96 N = 1
**PATIENT BURDEN**	Flesch-Kincaid Grade	4.8	12
	**Time to complete**	<1 minute	<1 minute
**ADMIN BURDEN**	Scoring	By hand, using a ruler	By hand
	Resource required	Cannot be administered verbally or by phone.	
	Licensing	Free	Free
	Other	Photocopying the scale may change the length of the 10-cm line	
**OTHER**	Trials	RA, PsA, JIA, SpA	RA, JIA, SpA
	Validity evidence	RA	RA, JIA
	**# of validation papers**	18	10
	**Validated language versions**		
	**Translations**	n/a	n/a

FATIGUE			
GENERIC INFO	Name	VAS	NRS
	Full name	Visual analogue scale	Numerical rating scale
	Description	A continuous scale, usually presented as a 100 mm horizontal line, anchored by two verbal descriptors, representing opposing extremes of the measured health state Respondents are asked to mark the location along this line that best represents their level of the measured health state	A numerical rating scale in which a patient selects the whole number (0–10 integers) that best reflects the intensity of the measured trait
	# of items	1	1
	Scale type	Most commonly anchored by “no fatigue” (score of 0) and “extreme fatigue ”(score of 100 [100-mm scale])	An 11-point numeric scale (NRS 11) with 0 representing one extreme (e.g., “no fatigue”) and 10 representing the other pain extreme (e.g., “extreme fatigue”)
	Recall period	Varies, but most commonly 1 day	Varies, but most commonly 1 day
	Scoring	Using a ruler, the score is determined by measuring the distance (mm) on the 10-cm line between the lower anchor (0 mm) and the patient's mark, providing a range of scores from 0–100	The number that the respondent indicates on the scale is recorded.
RELIABLITY	Test-retest	0.79 (0.70-0.92) N=3	?
	Internal consistency	n/a	n/a
VALIDITY	Construct validity	100% N=1	100% N=1
RESPONSIVENESS	(Ability to detect change)	100% N=3	100% N=2
IRT		n/a	n/a
Paper/electronic agreement (ICC)		0.77 N = 1	0.84 N = 1
PATIENT BURDEN	Flesch-Kincaid Grade		7.6
	**Time to complete**	n/a	n/a
ADMIN BURDEN	Scoring	By hand, using ruler	By hand
	Resource required	Cannot be administered verbally or by phone.	
	Licensing	Free	Free
	Other	Photocopying the scale may change the length of the 10-cm line	
OTHER	Trials	RA, PsA	PsA, JIA, SpA
	Validity evidence	RA, PsA, SpA	
	**# of validation papers**	8	5
	**Validated language versions**		
	**Translations**		

FATIGUE

	Name	Facit-F	BRAF-MD
GENERIC INFO	Full name	Functional Assessment of Chronic Illness Therapy-Fatigue	Bristol Rheumatoid Arthritis Fatigue Multidimensional Questionnaire
	Description	developed to measure fatigue in oncology patients with anemia and is a stand-alone (or add-on) questionnaire in the Functional Assessment in Cancer Therapy measurement system. This has since been widened to include assessment of chronic illnesses (FACIT measurement system). T	The BRAF MDQ was developed to assess the overall experience and impact of RA fatigue, and its different dimensions
	# of items	13	20
	Scale type	5 responses from "Not at all" to "Very much	Four options from "Not at all," "A little," "Quite a bit," to "Very much," except for the first 3 items, which are numerical or categorical as appropriate (e.g., how many days did you experience fatigue in the past 7 days? 0 –7).
	Recall period	1 week	1 week
	Scoring	Items scored 0 – 4, with 2 positively phrased items reverse scored. Items are summed, multiplied by 13, then divided by the number of items actually answered, therefore allowing for missing items. However, more than 50% of items must be answered (i.e., at least 7 items).	Items scores are summed to provide a total fatigue score, including 4 subscale scores for physical fatigue (4 items), living with fatigue (7 items), cognitive fatigue (5 items), and emotional fatigue (4 items).
**RELIABLITY**	Test-retest	0.95 N=1	?
	Internal consistency	0.88 (0.82-0.96) N=3	0.92 N=1
**VALIDITY**	Construct validity	80% N=1	100% N=1
**RESPONSIVENESS**	(Ability to detect change)	100% N=3	100% N=1
**IRT**		?	DIF by age & sex, Measurement precision (information functions) N =1
**Paper/electronic agreement (ICC**		0.94 (0.93 – 0.96) N = 2	?
**PATIENT BURDEN**	Flesch-Kincaid Grade	2.8	5.2
	**Time to complete**	3-4 min	4-5min
**ADMIN BURDEN**	Scoring	By hand	By hand
	Resource required		
	Licensing	English versions are free to use, fees are typically waived for investigator-initiated studies.	Free
	Other		
**OTHER**	Trials	RA, SpA	RA
	Validity evidence	RA, SpA, PsA	RA
	**# of validation papers**	5	4
	**Validated language versions**		
	**Translations**	>50	6

Copyright © 201[illegible] by the International Consortium for Health Outcomes Measurement. All rights reserved.

FATIGUE

	Name	FAS	PROMIS Fatigue CAT
GENERIC INFO	Full name	Fatigue Assessment Scale	
	Description	Unidimensional , generic Fatigue instrument	The PROMIS Fatigue instruments evaluate a range of self-reported symptoms, from mild subjective feelings of tiredness to an overwhelming, debilitating, and sustained sense of exhaustion that likely decreases one's ability to execute daily activities and function normally in family or social roles
	# of items	10	varies
	Scale type	5 options ranging from "never" to always"	5 response options ranging in value from 1 to 5
	Recall period	Present tense	1 week
	Scoring	A total FAS score is obtained by summing all 10 items.	Scoring function based on graded response model.
**RELIABLITY**	Test-retest	?	?
	Internal consistency	?	0.98 N=1
**VALIDITY**	Construct validity	?	?
**RESPONSIVENESS**	(Ability to detect change)	?	75% N=1
**IRT**		?	?
**Paper/electronic agreement (ICC**		?	?
**PATIENT BURDEN**	Flesch-Kincaid Grade	4.5	4.9
	**Time to complete**	n/a	n/a
**ADMIN BURDEN**	Scoring	By hand	IRT based score calculation
	Resource required		IT system in place
	Licensing	Free	Via assessment center (fee payable)
	Other		
**OTHER**	Trials		
	Validity evidence		RA
	**# of validation papers**		1
	**Validated language versions**		
	**Translations**	2	Dutch, English, Portuguese , Spanish

Copyright © 2017 by the International Consortium for Health Outcomes Measurement. All rights reserved.

HRQoL (pediatric)

	Name	PedsQL 4.0 Fatigue total score	General fatigue	Sleep rest	Cognitive fatigue
GENERIC INFO	Full name	Pediatric Quality of Life Inventory-Multidimensional Fatigue Scale			
	Description	the PedsQL 3.0 measurement model assesse 5 dimensions of health related quality of life in children using developmentally appropriate scales for children aged 5-7, 8-12. 13.18. Parent proxy versions are also available. The number of items per scale differs by age version for some subscales.			
	# of items	18	6	6	6
	Scale type	5-point Likert scale from 0 (Never) to 4 (Almost always) 3-point scale: 0 (Not at all), 2 (Sometimes) and 4 (A lot) for the Child Report for Young Children (ages 5-7)			
	Recall period				
	Scoring	items are reversed scored and linearly transformed to a 0-100 scale as follows: 0=100, 1=75, 2=50, 3=25, 4=0			
RELIABLITY	Test-retest	?	?	?	
	Internal consistency	0.78 (child); 0.86 (proxy)	0.783(child); 0.78 (proxy)	0.67 (child); 0.71 (proxy)	0.72 (child); 0.83 (proxy)
VALIDITY	Construct validity	?	?	?	
RESPONSIVENESS	(Ability to detect change)	?	?	?	
IRT		?	?	?	?
Paper/electronic agreement (ICC		?	?	?	?
PATIENT BURDEN	Flesch-Kincaid Grade	?	?	?	? ? ?
	Time to complete	Not stated			
ADMIN BURDEN	Scoring	With a hand calculator			
	Resource required				
	Licensing	License fee required for funded academic use			
	Other				
OTHER	Trials	JIA			
	Validity evidence	JIA			
	# of validation papers	1			
	Validated language versions	1			
	Translations	6			

Copyright © 2017 by the International Consortium for Health Outcomes Measurement. All rights reserved.

PATIENT / PARENT GLOBAL ASSESSMENT

GENERIC INFO	**Name**	**VAS**
	Full name	Visual analogue scale
	Description	A continuous scale, usually presented as a 100 mm horizontal line, anchored by two verbal descriptors, representing opposing extremes of the measured health state Respondents are asked to mark the location along this line that best represents their level of the measured health state
	# of items	1
	Scale type	May be anchored at the ends (e.g., : very well to very poor) or open and may have periodic tick marks at specified intervals
	Recall period	Varies, but most commonly 1 day
	Scoring	Using a ruler, measure in mm from the left border of the VAS to the point where the patient marked their response on the line. VAS consisting of circles may be scored by visual inspection without use of a ruler
**RELIABLITY**	Test-retest	0.84 (0.75 - 0.93) N = 2
	Internal consistency	n/a
**VALIDITY**	Construct validity	?
**RESPONSIVENESS**	(Ability to detect change)	100% N = 10
**IRT**		n/a
**Paper/electronic agreement (ICC**		0.84 (0.75 - 0.98) N = 3
**PATIENT BURDEN**	Flesch-Kincaid Grade	10.2
	**Time to complete**	10 seconds
**ADMIN BURDEN**	Scoring	By hand, using a ruler
	Resource required	Cannot be administered verbally or by phone.
	Licensing	Free
	Other	Photocopying the scale may change the length of the 10-cm line
**OTHER**	Trials	RA, PsA, JIA, SpA
	Validity evidence	RA, PsA, JIA, SpA
	**# of validation papers**	21
	**Validated language versions**	
	**Translations**	n/a

Copyright © 2017 by the International Consortium for Health Outcomes Measurement. All rights reserved.

PHYSICAL FUNCTION			
GENERIC INFO	Name	HAQ-DI	MHAQ
	Full name	Health Assessment Questionnaire Disability Index	Modified Health Assessment Questionnaire
	Description	The HAQ Disability index measures difficulty in performing activities of daily living in 8 categories of function. It is the most widely used disability instrument in rheumatology. It was originally developed for use in patients with arthritis, but has subsequently been used in a variety of settings.	The MHAQ is a modified version of the HAQ including only activity of each HAQ category. Activities were selected on the basis of intuitive choice of which 1 of the 2 or 3 in each HAQ category was most likely to apply to the largest number of people.
	# of items	20	8
	Scale type	Rating scale ranging from 0 (without any difficulty) to 3 (unable to do)	Rating scale ranging from 0 (without any difficulty) to 3 (unable to do)
	Recall period	1 week	1 week
	Scoring	20 items grouped in 8 categories: Dressing (2), rising (2), eating (2), walking (2), hygiene (3), reaching (3), Grip (2) activities (3). HAQ score is obtained by averaging 8 category scores (category score = item with highest level of reported disability). Category scores can be adjusted for the use of help and aids	Mean of 8 items
RELIABLITY	Test-retest	0.93 (0.81 - 0.99) N=8	0.89 (0.89 – 0.90) N=3
	Internal consistency	0.94 (0.86 - 0.94) N=18	0.87 (081-0.90) N=3
VALIDITY	Construct validity	91% (80 – 100%) N=7	80% N=1
RESPONSIVENESS	(Ability to detect change)	100% N=10	100% N=5
IRT		Measurement precision, Common metric (PF10 and promis), DIF by sex, age, diagnosis, disease duration, sacroiliitis), Person separation, fit to the Rasch model, Fit to Generalized partial credit model, unidimensionality, rating scale functioning, (N = 9)	?
Paper/electronic agreement (ICC		0.96 & 0.97 N = 2	?
PATIENT BURDEN	Flesch-Kincaid Grade	2.9	2.9
	**Time to complete**	<10 minutes	<5 minutes
ADMIN BURDEN	Scoring	By hand	By hand
	Resource required		
	Licensing	Free	Free
	Other		
OTHER	Trials	RA, PsA	RA, PsA
	Validity evidence	RA, SpA, PsA	RA
	**# of validation papers**	81	18
	**Validated language versions**	Arabic, Bengali, British, Chinese, Danish, Dutch	
	**Translations**	60 +	

PHYSICAL FUNCTION

	Name	HAQ-S	BASFI
GENERIC INFO	Full name	Health Assessment Questionnaire modified for spondyloarthropathies (function-S)	Bath Ankylosing Spondylitis Functional Activity Index
	Description	HAQ-S is a modified versions of the HAQ. It includes the 20 original HAQ items and additionally includes 5 activities important to patients with AS	Used to define and monitor functional ability in persons with ankylosing spondylitis.
	# of items	25	10
	Scale type	Rating scale ranging from 0 (without any difficulty) to 3 (unable to do)	10-cm visual analog scale, ranging from 0 (easy) to 10 (impossible)
	Recall period	1 week	1 week
	Scoring	20 HAQ items plus 5 questions specific to those persons with spondylitis about driving a car in reverse, using the rear view mirror in the car, carrying a full grocery bag, sitting for long periods, and working at a desk	The mean of the 10 scales gives the BASFI score (0 –10).
**RELIABLITY**	Test-retest	0.89 N=1	0.91 (0.68 – 0.99) N=18
	Internal consistency	0.91 N=1	0.91 (0.75 – 0.98) N=24
**VALIDITY**	Construct validity	?	?
**RESPONSIVENESS**	(Ability to detect change)	?	100% N=6
**IRT**		?	Fit to Rasch model, rating scale functioning, DIF N = 1
**Paper/electronic agreement (ICC**		?	0.95 & 0.9 N = 2
**PATIENT BURDEN**	Flesch-Kincaid Grade	4	6.7
	**Time to complete**	Not stated	<100 sec
**ADMIN BURDEN**	Scoring	By hand	By hand
	Resource required		
	Licensing	Free	Free
	Other		
**OTHER**	Trials	SpA	SpA , JIA, PsA
	Validity evidence	SpA	SpA, JIA
	**# of validation papers**	10	40
	**Validated language versions**	Brazilian-Portuguese, Hindi, Turkish	UK, Dutch, Brazilian-Portuguese, Indian, Iranian, Ukrainian, Moroccan, Tunisian, Chinese, Croatian, Arabic, Greek, Russian, Danish, Taiwan, Italian, Turkish, Spanish, Swedish, German, Portuguese, Lithuanian, Thai Czech
	**Translations**		76

Copyright © 2017 by the International Consortium for Health Outcomes Measurement. All rights reserved.

PHYSICAL FUNCTION

	Name	C-HAQ	HAQ-II
GENERIC INFO	Full name	Childhood Health Assessment Questionnaire	Health Assessment Questionnaire Two
	Description	Disability index adapted from the Stanford HAQ for use in children by adding several new questions such that for each functional area, there is at least 1 question that is relevant to children of all ages	Unidimensional scale that intends to maximize measurement precision across a wider range of disability (particularly with respect to higher levels of function) than the original HAQ-DI. Was developed using Rasch analyses to achieve this.
	# of items	30	10
	Scale type	Rating scale ranging from 0 (without any difficulty) to 3 (unable to do)	Rating scale ranging from 0 (without any difficulty) to 3 (unable to do)
	Recall period	1 week	1 week
	Scoring	See HAQ-DI	Mean of 10 items
RELIABLITY	Test-retest	0.88 (0.85-0.91) N=2	?
	Internal consistency	0.95 (0.88-0.99) N=33	0.88 N=2
VALIDITY	Construct validity	80% N=1	100% N=1
RESPONSIVENESS	(Ability to detect change)	100 % N=4	?
IRT		Fit to Rasch model, DIF by age N = 1	DIF by language version, sex, age and disease duration, person separation, fit to Rasch model N = 3
Paper/electronic agreement (ICC		?	?
PATIENT BURDEN	Flesch-Kincaid Grade	3.9	4
	Time to complete	10 minutes	n/a
ADMIN BURDEN	Scoring	By hand	By hand
	Resource required		
	Licensing	Free	Free
	Other		
OTHER	Trials	JIA	
	Validity evidence	JIA	RA
	# of validation papers		
	Validated language versions	Arabic, Argentinian, Austrian, Belgian-Flemish, Brazilian, British, Bulgarian, Chilean, Cost-Rican, Croatian, Czech, Danish, Dutch, European Spanish, French, Georgian, German, Greek, Hebrew, Hungarian, Italian, Japanese, Korean, Latvian, Mexican, Moroccan, Norwegian, Polish, Portuguese, Russian, Russian, Serbian, Slovak, Spanish, Swedish, Swiss German, Swiss French, Turkish	US, Dutch, French
	Translations	?	

PHYSICAL FUNCTION

	Name	PROMIS SF	PROMIS V 1.0 CAT
GENERIC INFO	Full name	Patient reported outcome measurement information system physical function	Patient reported outcome measurement information system physical function
	Description	a generic Physical Function item bank to be used across diseases and different levels of ability. Items are adapted from previously validated questionnaires	a generic Physical Function item bank to be used across diseases and different levels of ability. Items are adapted from previously validated questionnaires
	# of items	10	varies
	Scale type	Rating scale ranging from 1 (without any difficulty) to 5 (unable to do)	Rating scale ranging from 1 (without any difficulty) to 5 (unable to do)
	Recall period	1 day	1 day
	Scoring	Scoring function based on graded response model. Alternatively, tables are available to convert raw summed scores to approximate T-scores.	Scoring function based on Graded response model (IRT)
**RELIABLITY**	Test-retest	?	?
	Internal consistency	?	?
**VALIDITY**	Construct validity	50% N=1	?
**RESPONSIVENESS**	(Ability to detect change)	66% N=1	?
**IRT**		Common metric (HAQ-DI+PF10) DIF by sex, condition, age N = 2	Measurement precision (but not with official PROMIS calibration) N = 2
**Paper/electronic agreement (ICC**		?	?
**PATIENT BURDEN**	Flesch-Kincaid Grade	4.3	4.3
	**Time to complete**	n/a	~100 sec
**ADMIN BURDEN**	Scoring	Manually or dedicated software is required	IRT based score calculation requires access to dedicated software
	Resource required		Computer and internet access
	Licensing	Paper version – free	Via Assessment Centre (fee payable for all use)
	Other		
**OTHER**	Trials		
	Validity evidence	RA	RA
	**# of validation papers**	2	1
	**Validated language versions**	US, Dutch	US, Dutch
	**Translations**	6	6

Copyright © 2017 by the International Consortium for Health Outcomes Measurement. All rights reserved.

HRQoL

	Name	SF-36 PF10	RP	BP	GH	VT	SF	RE	MH
GENERIC INFO	Full name	Medical outcome study short form 36							
	Description	the SF-36 intends to measure "general health concepts not specific to any age, disease, or treatment group							
	# of items	10	4	2	5	4	2	3	5
	Scale type	The response scales for the SF-36 items vary across and within the scales, with the number of response options ranging from 3 (physical functioning) to 6 (vitality and mental health). The health transition item is scored on a 5-point scale where 1 indicates much better than a year ago, and 5 indicates much worse than a year ago.							
	Recall period	4 & 1 week versions							
	Scoring	scale scores are calculated by summing responses across scale items and then transforming these raw scores to a 0–100 scale							
**RELIABLITY**	Test-retest	0.90 (0.88-0.93) N=4	0.85 N=4	0.79 N=5	0.84 N=4	0.85N=4	0.65 N=4	0.70 N=4	0.76 N=5
	Internal consistency	0.92(0.89-0.94) N-8	0.87 N=6	0.87 N=6	0.77 N=6	0.82 N=6	0.83 N=6	0.84 N=6	0.8 N=6
**VALIDITY**	Construct validity	80% (0-100%) N=7	78% N=5	95% N=6	98% N=5	96% N=7	90% n=5	87% N=4	89% N=4
**RESPONSIVENESS**	(Ability to detect change)	93% N=15	100% N=8	100% N=9	87.5% N=8	100% N=8	100% N=9	87.5% N=8	87.5% N=8
**IRT**		Measurement precision, common metric with PROMIS, HAQ. Fit to rasch model, unidimensionality, person separation, DIF by sex, age, sacroiliitis, diagnosis. Rating scale functioning N = 5				Measurement precision DIF by sex and age N = 1			
**Paper/electronic agreement (ICC**		?	?	?	?	?	?	?	?
**PATIENT BURDEN**	Flesch-Kincaid Grade	4.4	9	7	3.7	4.4	14.2	9.9	4.4
	**Time to complete**	5 minutes (Sf-36 v2 total)							
**ADMIN BURDEN**	Scoring	computerized scoring							
	Resource required				need computer access for scoring				
	Licensing	License required for non-academic use							
	Other								
**OTHER**	Trials	RA, SpA, PsA							
	Validity evidence	RA, SpA, PsA							
	**# of validation papers**	37							
	**Validated language versions**	4							
	**Translations**	121							

Copyright © 2017 by the International Consortium for Health Outcomes Measurement. All rights reserved.

HRQoL			
GENERIC INFO	**Name**	**EQ-5D**	**SF-6D**
	Full name	Euroqol 5 dimensions	Medical outcome study short form 6 D
	Description	The EQ-5D is a generic measure of health status for use in clinical, population, and economic appraisals with adult samples. The measure was developed by the European Quality of Life Group (EuroQol) to act as a core set of items for use in international studies measuring health-related quality of life across a wide range of health conditions and treatments. It provides a simple descriptive profile and single index values of health status.	The purpose of the SF-6D is to provide ratings of an individual's health-related quality of life (HRQOL) across all health conditions. The ratings of HRQOL are also called "utilities" or preferences for health states that are used in health economic evaluation and to derive quality-adjusted life years (QALYs) for use in cost utility analysis
	# of items	5	11
	Scale type	3 & 5 point rating scales ranging from I have no/am not to I am unable to/have extreme	The response scales for the items vary across and within the scales, with the number of response options ranging from 3 (physical functioning) to 6 (vitality and mental health). The health transition item is scored on a 5-point scale where 1 indicates much better than a year ago, and 5 indicates much worse than a year ago.
	Recall period	1 day	4 & 1 week versions
	Scoring	The EQ-5D Index uses a utility-weighted scoring system that has been derived from extensive studies with different countries or by taking into account an individual's own preferences as reflected in the VAS rating scale from 0–100. A constant is also subtracted if one or more dimensions are scored at 2 or 3, and a further constant if one or more dimensions are scored at 3. A negative score is possible in creating the EQ-5D Index, representing a state "worse than death." The EQ-5D was developed using health economics principles.	The SF-6D utility score is calculated as a function of weighted scores across the items that comprise this tool
**RELIABLITY**	Test-retest	0.75 (0.66-0.83) N=4	0.72 N=1
	Internal consistency	0.84 N=1	0.83 N=1
**VALIDITY**	Construct validity	70% (50-100%) N=4	100% N=1
**RESPONSIVENESS**	(Ability to detect change)	75% N=8	75% N=13
**IRT**		n/a	n/a
**Paper/electronic agreement (ICC**		0.79 & 0.93 N = 2	?
**PATIENT BURDEN**	Flesch-Kincaid Grade	6	9
	**Time to complete**	<2min	n/a
**ADMIN BURDEN**	Scoring	computerized scoring	computerized scoring
	Resource required	Time and training are needed to score the EQ-5D. The EuroQol web site must be consulted to register studies and to determine the appropriate rates for a country.	Training required for score interpretation and need computer access for scoring
	Licensing	License required for commercial use	License required for commercial use
	Other		
**OTHER**	Trials	RA, SpA, PsA	RA, SpA, PsA
	Validity evidence	RA, SpA, PsA	RA, SpA, PsA
	**# of validation papers**	22	14
	**Validated language versions**		

HRQoL (pediatric)

GENERIC INFO	Name	PedsQL 3.0 Aches and pain	Daily activities	Treatment	Worry	communication
	Full name	Pediatric Quality of Life Inventory - PedsQL TM				
	Description	the PedsQL 3.0 measurement model assesse 5 dimensions of health related quality of life in children using developmentally appropriate scales for children aged 5-7, 8-12. 13.18. Parent proxy versions are also available. The number of items per scale differs by age version for some subscales.				
	# of items	4	5	5-7	3	3
	Scale type	The response scales for the SF-36 items vary across and within the scales, with the number of response options ranging from 3 (physical functioning) to 6 (vitality and mental health). The health transition item is scored on a 5-point scale where 1 indicates much better than a year ago, and 5 indicates much worse than a year ago.				
	Recall period	4 & 1 week versions				
	Scoring	Items are reversed scored and linearly transformed to a 0-100 scale Mean score = Sum of the items over the number of items answered. f more than 50% of the items in the scale are missing, the Scale scores should not be computed. If 50% or more items are completed: Impute the mean of the completed items in a scale.				
RELIABLITY	Test-retest	?	?	?	?	
	Internal consistency	0.86 (child) 0.91 (proxy)	0.78child) 0.91 (proxy)	0.80child) 0.82 (proxy)	0.75child) 0.83 (proxy)	0.78child) 0.89 (proxy)
VALIDITY	Construct validity	?	?	?	?	?
RESPONSIVENESS	(Ability to detect change)	?	?	?	?	?
IRT		?	?	?	?	?
Paper/electronic agreement (ICC		?	?	?	?	?
PATIENT BURDEN	Flesch-Kincaid Grade	?	?	?	?	? ?
	Time to complete	Not stated				
ADMIN BURDEN	Scoring	With a hand calculator				
	Resource required	need calculator for scoring				
	Licensing	License fee required for funded academic use				
	Other					
OTHER	Trials	JIA				
	Validity evidence	JIA				
	# of validation papers	1				
	Validated language versions	1				
	Translations	6				

Copyright © 2017 by the International Consortium for Health Outcomes Measurement. All rights reserved.

HRQoL (pediatric)

	Name	PedsQL 4.0 total	Physical function	Emotional function	Social function	School function			
GENERIC INFO	Full name	Pediatric Quality of Life Inventory - PedsQL TM							
	Description	the PedsQL 4.0 measurement model assesse 4 dimensions of health related quality of life in children using developmentally appropriate scales for children aged 5-7, 8-12. 13.18. Parent proxy versions are also available. The number of items per scale differs by age version for some subscales.							
	# of items	23	8	5	5	5			
	Scale type	The response scales for the SF-36 items vary across and within the scales, with the number of response options ranging from 3 (physical functioning) to 6 (vitality and mental health). The health transition item is scored on a 5-point scale where 1 indicates much better than a year ago, and 5 indicates much worse than a year ago.							
	Recall period	4 & 1 week versions							
	Scoring	Items are reversed scored and linearly transformed to a 0-100 scale Mean score = Sum of the items over the number of items answered. f more than 50% of the items in the scale are missing, the Scale scores should not be computed. If 50% or more items are completed: Impute the mean of the completed items in a scale.							
RELIABLITY	Test-retest	?	?	?	?	?	?		
	Internal consistency	0.85 (5-7 years), 0.92 (8-12), 0.92 (13-18)	?	?	?	?	?		
VALIDITY	Construct validity	?	?	?	?	?	?		
RESPONSIVENESS	(Ability to detect change)	?	?	?	?	?	?		
IRT		?	?	?	?	?	?		
Paper/electronic agreement (ICC		?	?	?	?	?	?		
PATIENT BURDEN	Flesch-Kincaid Grade	(8-12 years version)	1.7	1.4	2.0	2.5	1.4	2.0	2.5
	**Time to complete**	Not stated							
ADMIN BURDEN	Scoring	With a hand calculator							
	Resource required	need calculator for scoring							
	Licensing	License fee required for funded academic use							
	Other								
OTHER	Trials								
	Validity evidence								
	**# of validation papers**	1							
	**Validated language versions**	0							
	**Translations**	111							

Copyright © 2017 by the International Consortium for Health Outcomes Measurement. All rights reserved.

HRQoL (pediatric)

GENERIC INFO	Name	CHQ
	Full name	Child Health Questionnaire
	Description	The Child Health Questionnaire™ (CHQ) is a family of general pediatric quality of life surveys that have been designed and normed for children from 5-to-18 years of age. his measure consists of child report (ages 10–18 years) and 2 versions of parent-proxy report (ages 5–18 years) of the child's HRQOL. It can be used with healthy children and those with both acute and chronic health conditions. Assesses for 14 physical and psychosocial domains: general health perceptions, physical functioning, role/social physical functioning, bodily pain, role/social emotional functioning, role/social behavioral functioning, parent impact-time, parent impact-emotional, self-esteem, mental health, behavior, family activities, family cohesion, and change in health.
	# of items	The child-report questionnaire (CHQ-CF87) consists of 87 items. The long parent-report questionnaire (CHQ-PF50) consists of 50 items, and the short parent-report questionnaire (CHQ-PF28) consists of 28 items.
	Scale type	response options for the CHQ are ordinal scales that vary by the item. Each item consists of 4–6 response options. Additionally, each scale consists of varying numbers of items.
	Recall period	Varies by subscale. Most scales have a recall period of 4 weeks.
	Scoring	Overall means for the individual CHQ scales and items can be derived using a simple summated rating approach. This method yields a profile for each of the 14 health concepts. In addition, the individual scale scores can be aggregated to derive 2 summary component scores: the physical functioning and psychosocial health summary scores. Scores are transformed to a 0–100 scale, with a mean ± SD of 50 ± 10. The CHQ Scoring and Interpretation Manual is available on CD and is required for scoring and interpretation.
**RELIABLITY**	Test-retest	?
	Internal consistency	0.94 (0.92-0.97) N=33 (total score)
**VALIDITY**	Construct validity	?
**RESPONSIVENESS**	(Ability to detect change)	?
**IRT**		?
**Paper/electronic agreement (ICC**		?
**PATIENT BURDEN**	Flesch-Kincaid Grade	4.0
	**Time to complete**	14.5 minutes
**ADMIN BURDEN**	Scoring	Using a calculator
	Resource required	
	Licensing	Free
	Other	m response options and recall periods vary by item and the number of items is very large.
**OTHER**	Trials	JIA
	Validity evidence	JIA
	**# of validation papers**	
	**Validated language versions**	Arabic, Argentinian, Austrian, Belgian-Flemish, Brazilian, British, Bulgarian, Chilean, Cost-Rican, Croatian, Czech, Danish, Dutch, European Spanish, French, Georgian, German, Greek, Hebrew, Hungarian, Italian, Japanese, Korean, Latvian, Mexican, Moroccan, Norwegian, Polish, Portuguese, Russian, Russian, Serbian, Slovak, Spanish, Swedish, Swiss German, Swiss French, Turkish
	**Translations**	72

Copyright © 2017 by the International Consortium for Health Outcomes Measurement. All rights reserved.

HRQoL			
GENERIC INFO	**Name**	**PROMIS Global Health**	**MSK-HQ**
	Full name		
	Description	The 10 global health items include ratings of the five core PROMIS domains and ratings that cut across domains	
	# of items	10	15
	Scale type	5 point rating scale	5 point rating scale
	Recall period	Past 7 days & present tense	Past 2 weeks
	Scoring	Scoring function based on graded response model. Alternatively, tables are available to convert raw summed scores to approximate T-scores.	Summed score of 14 items
**RELIABLITY**	Test-retest	?	?
	Internal consistency	?	?
**VALIDITY**	Construct validity	?	?
**RESPONSIVENESS**	(Ability to detect change)	?	?
**IRT**		?	?
**Paper/electronic agreement (ICC**		?	?
**PATIENT BURDEN**	Flesch-Kincaid Grade	8,4	9,6
	**Time to complete**	?	?
**ADMIN BURDEN**	Scoring	IRT scores require dedicated software, conversion tables are available that approximate IRT scores	By hand
	Resource required	Access to assessment center for IRT scores ($ 5.000 per year per study)	
	Licensing	Paper version – free	Free for non-commercial use
	Other		
**OTHER**	Trials		
	Validity evidence		
	**# of validation papers**	0	0
	**Validated language versions**	0	0
	**Translations**	8	0

Copyright © 2017 by the International Consortium for Health Outcomes Measurement. All rights reserved.

HRQoL			
GENERIC INFO	**Name**	**PsAID (12 items version)**	**RAID**
	Full name		
	Description	PsAID is a patient reported and patient derived composite score to assess overall impact of psoriatic arthritis , combining information on 7 domains.	RAID is a patient reported and patient derived composite score to assess overall impact of RA, combining information on 7 domains.
	# of items	12	7 domains represented by one NRS each
	Scale type	0-10 Numerical rating scale	0-10 Numerical rating scale
	Recall period		Past week
	Scoring		Weighted summed score
**RELIABLITY**	Test-retest	> 0.94 N=1	0,90 N = 1
	Internal consistency	> 0.3 N=1	0,91 & 0,93 N = 2
**VALIDITY**	Construct validity	?	?
**RESPONSIVENESS**	(Ability to detect change)	100% N = 1	100% N = 1
**IRT**		?	?
**Paper/electronic agreement (ICC**		?	?
**PATIENT BURDEN**	Flesch-Kincaid Grade	12,5	10,5
	**Time to complete**	?	?
**ADMIN BURDEN**	Scoring	Using online calculator	Using online calculator
	Resource required		
	Licensing	Free	Free
	Other		
**OTHER**	Trials		
	Validity evidence		RA
	**# of validation papers**	2	3
	**Validated language versions**		
	**Translations**	12	67

WORK DISABILITY AND PRODUCTIVITY: PRESENTEEISM			
GENERIC INFO	Name	WPAI	WPS-RA
	Full name	Measures the effect of health and symptom severity on work productivity and nonwork activities.	Rheumatoid arthritis specific work productivity survey
	Description	WPAI presenteeism assesses impairment at work/ reduced on the job effectiveness	Measures the impact of rheumatoid arthritis (RA) on the productivity of employment work, household work, and daily activities.
	# of items	1	1
	Scale type		count of days missed
	Recall period	7 days	1 month
	Scoring		the number of days with productivity reduced by at least half is recorded
RELIABLITY	Test-retest	0.74 N=1	0.74 N=1
	Internal consistency	N/A	N/A
VALIDITY	Construct validity	100% N=1	100% N=1
RESPONSIVENESS	(Ability to detect change)	100% N=1	100% N=1
IRT		n/a	n/a
Paper/electronic agreement (ICC		?	?
PATIENT BURDEN	Flesch-Kincaid Grade	8.4	10
	Time to complete	n/a	n/a
ADMIN BURDEN	Scoring		By hand
	Resource required		
	Licensing		
	Other		
OTHER	Trials	RA	
	Validity evidence	RA, SpA	RA. SpA
	# of validation papers	7	3
	Validated language versions		
	Translations		

WORK DISABILITY AND PRODUCTIVITY: ABSENTEEISM

	Name	WPAI	WPS-RA
GENERIC INFO	Full name	Measures the effect of health and symptom severity on work productivity and nonwork activities.	Rheumatoid arthritis specific work productivity survey
	Description	Measures the effect of health and symptom severity on work productivity and nonwork activities	Measures the impact of rheumatoid arthritis (RA) on the productivity of employment work, household work, and daily activities.
	# of items	3	1
	Scale type		count of days missed
	Recall period		1 month
	Scoring		the number of missed days is recorded
RELIABLITY	Test-retest	?	?
	Internal consistency	n/a	n/a
VALIDITY	Construct validity	80% N=1	100% N=1
RESPONSIVENESS	(Ability to detect change)	100% N=1	100% N=1
IRT		n/a	n/a
Paper/electronic agreement (ICC		?	?
PATIENT BURDEN	Flesch-Kincaid Grade	8.6	5.9
	Time to complete	n/a	n/a
ADMIN BURDEN	Scoring		By hand
	Resource required		
	Licensing		
	Other		
OTHER	Trials	RA, SpA	RA, SpA
	Validity evidence	RA, SpA	RA, SpA
	# of validation papers	2	5
	Validated language versions		
	Translations		

references

1. Stucki, G., & Cieza, A. (2004). The International Classification of Functioning, Disability and Health (ICF) Core Sets for rheumatoid arthritis: a way to specify functioning. *Annals of the rheumatic diseases*, *63 Suppl 2*(suppl_2), ii40-ii45. doi:10.1136/ard.2004.028233
2. Lootens, C. C., & Rapoff, M. A. (2011). Measures of pediatric pain: 21-numbered circle Visual Analog Scale (VAS), E-Ouch Electronic Pain Diary, Oucher, Pain Behavior Observation Method, Pediatric Pain Assessment Tool (PPAT), and Pediatric Pain Questionnaire (PPQ). *Arthritis care & research*, (S11), S253-62. doi:10.1002/acr.20634
3. Hawker, G. A., Mian, S., Kendzerska, T., & French, M. (2011). Measures of adult pain: Visual Analog Scale for Pain (VAS Pain), Numeric Rating Scale for Pain (NRS Pain), McGill Pain Questionnaire (MPQ), Short-Form McGill Pain Questionnaire (SF-MPQ), Chronic Pain Grade Scale (CPGS), Short Form-36 Bodily Pain Scale (SF. *Arthritis Care & Research*, *63*(S11), S240–S252. doi:10.1002/acr.20543
4. Carle, A. C., Dewitt, E. M., & Seid, M. (2011). Measures of health status and quality of life in juvenile rheumatoid arthritis: Pediatric Quality of Life Inventory (PedsQL) Rheumatology Module 3.0, Juvenile Arthritis Quality of Life Questionnaire (JAQQ), Paediatric Rheumatology Quality of Life Scale (PRQL), and Childhood Arthritis Health Profile (CAHP). *Arthritis care & research*, (S11), S438-45. doi:10.1002/acr.20560
5. Hewlett, S., Dures, E., & Almeida, C. (2011). Measures of fatigue: Bristol Rheumatoid Arthritis Fatigue Multi-Dimensional Questionnaire (BRAF MDQ), Bristol Rheumatoid Arthritis Fatigue Numerical Rating Scales (BRAF NRS) for Severity, Effect, and Coping, Chalder Fatigue Questionnaire (CFQ), Checklist. *Arthritis Care & Research*, *63*(S11), S263–S286. doi:10.1002/acr.20579
6. Tang, K., Boonen, A., Verstappen, S. M. M., Escorpizo, R., Luime, J. J., Lacaille, D., … Beaton, D. E. (2013). Worker Productivity Outcome Measures: OMERACT Filter Evidence and Agenda for Future Research. *The Journal of Rheumatology*, *41*(1), 165–176. doi:10.3899/jrheum.130815
7. Maska, L., Anderson, J., & Michaud, K. (2011). Measures of functional status and quality of life in rheumatoid arthritis: Health Assessment Questionnaire Disability Index (HAQ), Modified Health Assessment Questionnaire (MHAQ), Multidimensional Health Assessment Questionnaire (MDHAQ), Health Assessment. *Arthritis Care & Research*, *63*(S11), S4–S13. doi:10.1002/acr.20620
8. Zochling, J. (2011). Measures of symptoms and disease status in ankylosing spondylitis: Ankylosing Spondylitis Disease Activity Score (ASDAS), Ankylosing Spondylitis Quality of Life Scale (ASQoL), Bath Ankylosing Spondylitis Disease Activity Index (BASDAI), Bath Ankylosing Sp. *Arthritis Care & Research*, *63*(S11), S47–S58. doi:10.1002/acr.20575
9. Klepper, S. E. (2011). Measures of pediatric function: Child Health Assessment Questionnaire (C-HAQ), Juvenile Arthritis Functional Assessment Scale (JAFAS), Pediatric Outcomes Data Collection Instrument (PODCI), and Activities Scale for Kids (ASK). *Arthritis care & research*, (S11), S371-82. doi:10.1002/acr.20635
10. Tang, K., Beaton, D. E., Boonen, A., Gignac, M. A. M., & Bombardier, C. (2011). Measures of work disability and productivity: Rheumatoid Arthritis Specific Work Productivity Survey (WPS-RA), Workplace Activity Limitations Scale (WALS), Work Instability Scale for Rheumatoid Arthritis (RA-WIS), Work Limitations Questionnaire (WLQ), and. *Arthritis Care & Research*, *63*(S11), S337–S349. doi:10.1002/acr.20633
11. Busija, L., Pausenberger, E., Haines, T. P., Haymes, S., Buchbinder, R., & Osborne, R. H. (2011). Adult measures of general health and health-related quality of life: Medical Outcomes Study Short Form 36-Item (SF-36) and Short Form 12-Item (SF-12) Health Surveys, Nottingham Health Profile (NHP), Sickness Impact Profile (SIP), Medical Outcomes Study Short Form 6D (SF-6D), Health Utilities Index Mark 3 (HUI3), Quality of Well-Being Scale (QWB), and Assessment of Quality of Life (AQoL). *Arthritis care & research*, (S11), S383-412. doi:10.1002/acr.20541
12. Jacobs, J. W., Oosterveld, F. G., Deuxbouts, N., Rasker, J. J., Taal, E., Dequeker, J., & Uytterhoeven, R. (1992). Opinions of patients with rheumatoid arthritis about their own functional capacity: how valid is it? *Annals of the Rheumatic Diseases*, *51*(6), 765--8.
13. White, D. K., Wilson, J. C., & Keysor, J. J. (2011). Measures of adult general functional status: SF-36 Physical Functioning Subscale (PF-10), Health Assessment Questionnaire (HAQ), Modified Health Assessment Questionnaire (MHAQ), Katz Index of Independence in activities of daily living, Functional Independence Measure (FIM), and Osteoarthritis-Function-Computer Adaptive Test (OA-Function-CAT). *Arthritis care & research*, (S11), S297-307. doi:10.1002/acr.20638
14. Gignac, M. A. M., Cao, X., Mcalpine, J., & Badley, E. M. (2011). Measures of disability: Arthritis Impact Measurement Scales 2 (AIMS2), Arthritis Impact Measurement Scales 2-Short Form (AIMS2-SF), The Organization for Economic Cooperation and Development (OECD) Long-Term Disability (LTD) Questionnaire, EQ-5D, World Health Organization Disability Assessment Schedule II (WHODASII), Late-Life Function and Disability Instrument (LLFDI), and Late-Life Function and Disability Instrument-Abbreviated Version (LLFDI-Abbreviated). *Arthritis care & research*, (S11), S308-24. doi:10.1002/acr.20640
15. Anderson, J., Zimmerman, L., Caplan, L., & Michaud, K. (2011). Measures of rheumatoid arthritis disease activity: Patient (PtGA) and Provider (PrGA) Global Assessment of Disease Activity, Disease Activity Score (DAS) and Disease Activity Score with 28-Joint Counts (DAS28), Simplified Disease Activity Index (SDAI), Cl. *Arthritis Care Res (Hoboken).*

Copyright © 2017 by the International Consortium for Health Outcomes Measurement. All rights reserved.

Copyright © 2017 by the International Consortium for Health Outcomes Measurement. All rights reserved.
